# TMEM30A is essential for hair cell polarity maintenance in postnatal mouse cochlea

**DOI:** 10.1186/s11658-023-00437-w

**Published:** 2023-03-23

**Authors:** Yazhi Xing, Kun Peng, Qian Yi, Dongzhen Yu, Haibo Shi, Guang Yang, Shankai Yin

**Affiliations:** 1 Department of Otolaryngology Head & Neck Surgery, Shanghai Sixth People’s Hospital Affiliated to Shanghai Jiao Tong University School of Medicine, 1301 Research Bldg, 600 Yishan Rd, Shanghai, China; 2 Otolaryngology Institute of Shanghai Jiao Tong University, 600 Yishan Rd, Shanghai, 200233 China; 3 Health Management Center, Sichuan Provincial People’s Hospital, University of Electronic Science and Technology of China, Chengdu, 610054 Sichuan China

**Keywords:** P4-ATPase, Flippases, Planar cell polarity, ER stress, Hearing loss

## Abstract

**Background:**

Phosphatidylserine is translocated to the inner leaflet of the phospholipid bilayer membrane by the flippase function of type IV P-tape ATPase (P4-ATPase), which is critical to maintain cellular stability and homeostasis. Transmembrane protein 30A (TMEM30A) is the β-subunit of P4-ATPase. Loss of P4-ATPase function causes sensorineural hearing loss and visual dysfunction in human. However, the function of TMEM30A in the auditory system is unclear.

**Methods:**

P4-ATPase subtype expression in the cochlea was detected by immunofluorescence staining and quantitative real-time polymerase chain reaction (qRT-PCR) at different developmental stages. Hair cell specific TMEM30A knockout mice and wild-type littermates were used for the following functional and morphological analysis. Auditory function was evaluated by auditory brainstem response. We investigated hair cell and stereocilia morphological changes by immunofluorescence staining. Scanning electron microscopy was applied to observe the stereocilia ultrastructure. Differentially expressed transcriptomes were analyzed based on RNA-sequencing data from knockout and wild-type mouse cochleae. Differentially expressed genes were verified by qRT-PCR.

**Results:**

TMEM30A and subtypes of P4-ATPase are expressed in the mouse cochlea in a temporal-dependent pattern. Deletion of TMEM30A in hair cells impaired hearing onset due to progressive hair cell loss. The disrupted kinocilia placement and irregular distribution of spectrin-α in cuticular plate indicated the hair cell planar polarity disruption in TMEM30A deletion hair cells. Hair cell degeneration begins at P7 and finishes around P14. Transcriptional analysis indicates that the focal adhesion pathway and stereocilium tip-related genes changed dramatically. Without the TMEM30A chaperone, excessive ATP8A2 accumulated in the cytoplasm, leading to overwhelming endoplasmic reticulum stress, which eventually contributed to hair cell death.

**Conclusions:**

Deletion of TMEM30A led to disrupted planar polarity and stereocilia bundles, and finally led to hair cell loss and auditory dysfunction. TMEM30A is essential for hair cell polarity maintenance and membrane homeostasis. Our study highlights a pivotal role of TMEM30A in the postnatal development of hair cells and reveals the possible mechanisms underlying P4-ATPase-related genetic hearing loss.

**Supplementary Information:**

The online version contains supplementary material available at 10.1186/s11658-023-00437-w.

## Background

Hearing loss is the third most common disability in human and causes a major economic burden to patients and society. Genetic-related hearing loss accounts for over 50% of the neonatal population with hearing impairment [1] and nearly 40% of childhood hearing loss [2]. Over 250 genes are related to syndromic and nonsyndromic hearing loss [3]. Recently, ATP11A was reported as the gene related to DFNA33, a form of bilateral sensorineural hearing loss found in three Caucasian families [4, 5]. ATP8A2 is also required for normal visual and auditory function, while its mutation impedes the survival of photoreceptor and spiral ganglion neurons [6]. Patients with benign recurrent intrahepatic cholestasis type 1 (BRIC type 1) or progressive intrahepatic cholestasis type 1 (PEIC type 1) caused by mutations in ATP8B1 also present with progressive hearing loss, as observed in the ATP8B1 mutant mouse model [7]. ATP8A2, ATP8B1, and ATP11A belong to the Type IV P-tape ATPase (P4-ATPase) superfamily. These mutation related hearing losses in unrelated patients suggest that P4-ATPases play a critical role in the auditory system.

P4-ATPases are phospholipid flippases, used to translocate specific phospholipids, specifically phosphatidylserine (PS), from the exoplasmic leaflet to the cytoplasmic leaflet of membranes via the consumption of ATP. By generating and maintaining the asymmetry of the transmembrane lipid, P4-ATPases are essential for various cellular processes, including cell signaling, vesicle trafficking, apoptosis, bile and cholesterol homeostasis, and neuronal cell survival. There are fourteen subtypes of P4-ATPases in human, including ATP8A1, ATP8A2, ATP8B1-B4, ATP9A, ATP9B, ATP10B-10D, and ATP11A-11C. These subtypes are differentially expressed in various tissues. Dysfunction leads to the exposure of PS on the outer surface of the biological membrane, which acts as an “eat-me” signal inducing phagocytosis [8].

P4-ATPases require a β-subunit transmembrane protein 30 (TMEM30), also known as cell division control protein 50 (CDC50), to mediate the active transport of phospholipids across cellular membranes. Serving as a common yet irreplaceable subunit to form functional P4-ATPase heteromeric complexes, three isoforms have been found: TMEM30A, TMEM30B, and TMEM30C. TMEM30A is the most common form for P4-ATPases, except for ATP9A and ATP9B [9]. TMEM30A contributes to the protein stability and localization of the P4-ATPase flippase complex. In retina, deletion of TMEM30A caused vision dysfunction and a thinner layer of cone and rod cells [10, 11]. Cryoelectron microscopy analysis uncovered the yeast flippase Drs2p/CDC50p and ATP8A1/CDC50A structures and deciphered the possible ATPase reaction cycle [12, 13]. Localization of ATP8B1 requires TMEM30A for proper translocation from the endoplasmic reticulum (ER) to the plasma membrane. In the absence of TMEM30A, ATP8B1 aggregates in the ER. ATP11A localizes to the Golgi and plasma membrane when coexpressed with TMEM30A [14]. Lack of TMEM30A diminished flippase activity and led to PS exposure on the exoplasmic leaflet of the cellular membrane [15, 16].

The peripheral auditory perception system is located in the cochlea. The sensory receptor is mainly hair cells (HCs) that convert sound from mechanical vibration to electrical signals, and then spiral ganglion neurons (SGNs) transmit these signals to the central auditory systems. The HC function highly depends on the well-organized and highly maintained stereocilia, which are allocated on the cuticular plate at the apical surface of HCs. Disruption of stereocilia, and losses of HCs lead to hearing loss. Given the basic role as a β-subunit of the widely distributed P4-ATPases and evidence that deafness is inherited in patients with different subtype mutations, it is extremely important and reasonable to explore the function of TMEM30A to elucidate the role of P4-ATPases in the auditory system.

In this study, we investigated the expression pattern of P4-ATPases, including TMEM30A, in the postnatal and adult cochlea. By generating HC-specific TMEM30A knockout (KO) mice, we investigated functional and morphological alterations, as well as transcriptome changes in the cochleae. We showed that TMEM30A deletion caused disarrangement of the hair cell stereocilia and aberrant kinocilia at P7. Accumulation of P4-ATPases in hair cells further led to overwhelming ER stress, and finally caused hair cell loss and auditory dysfunction. Collectively, our study sheds light on the pivotal role of TMEM30A in the postnatal development and maturation of HCs in the postnatal cochlea.

## Methods and materials

### Animals

#### Generation of hair cell specific TMEM30A knockout mice

The animal study was reviewed and approved by the Animal Ethics Committee of Shanghai Jiao Tong University Affiliated Sixth People’s Hospital. The mice were maintained on a standard 12-h light, 12-h dark cycle and fed ad libitum with standard rodent chow. *Tmem30a*^loxp/loxp^ mice were kindly provided by Dr. Xianjun Zhu [10]. To assess the role of Tmem30a in hair cells, we crossed* Tmem30a*^loxp/loxp^ mice with Gfi-Cre and Atoh1-Cre mouse lines to generate hair cell specific TMEM30A knockout mice. Mice homozygous for the *Tmem30a* floxed allele (*Tmem30a*^loxp/loxp^) were mated with Gfi-Cre *Tmem30a*^loxp/+^ or Atoh1-cre *Tmem30a*^loxp/+^ mice to generate Gfi-Cre; *Tmem30a*^loxp/loxp^, Atoh1-Cre; *Tmem30a*^loxp/loxp^, and *Tmem30a*^loxp/loxp^ mice. In this study, *Tmem30a*^loxp/loxp^ mice were used as controls. Mice were genotyped according to standard PCR protocols from DNA extracted from mouse tail tips.

### Auditory function test

Auditory brainstem response tests (ABRs) were performed with the TDT sound-evoking system (Tucker-Davis Technology, USA) in a soundproof chamber at the assigned timepoint, which was at days 14, 21, 30, and 40 postnatal. The mice were anesthetized by intraperitoneal injection of 1% sodium pentobarbital (70 mg/kg), and their body temperature was maintained at approximately 37 °C with a thermostatic heating pad. The recording electrodes were placed under the skin at the vertex of the skull, and the reference and ground electrodes were positioned on each side of the mastoid. The speaker was 10 cm in front of the animal’s ears. The stimulus sounds were tone bursts (tone frequency 21.1/s, rise/fall time 0.5 ms, duration 10 ms, sampling rate 200 kHz) at 4, 8, 16, 32, and 40 kHz. The ABR-evoked potential was amplified 20 times by the preamplifier RA16 and collected (bandpass filter 300–3000 Hz, superposition times 1000, sampling rate 25 kHz). The stimulus started from 90 dB SPL and gradually decreased by 5 dB to 10 dB SPL. The last intensity that was recognizable as ABR wave III was defined as the hearing threshold. Here, 100 dB SPL was assigned when the animal showed no response to stimuli at 90 dB SPL for data collection.

### Whole mount preparation

After deep anesthetization, mice were decapitated. Cochleae were dissected, fixed with 4% paraformaldehyde through round window perfusion and immersed in the solution for 2 h at 4 °C. Basilar membranes were carefully dissected out as previously mentioned [17]. Whole basilar membranes were pretreated with 1% Triton in PBS for 1 h, followed by incubation with blocking solution for 1 h. The blocking solution was prepared with 5% normal goat serum and 1% Triton in PBS. Tissues were then incubated with primary antibody for 16 h at 4 °C. The second day, after three PBS washes, tissues were incubated with proper Alexa Fluor secondary antibodies for 3 h. After immersion in phalloidin-Fluor 555 (1:2000, Abcam, Cambridge, UK) for 30 min, tissues were mounted with antifade mounting medium (Vectashield, Burlingame, USA). Images were taken with a Zeiss 710 confocal microscope (Zeiss, Oberkochen, Germany) at 20× and 63× object magnifications. Images were processed and analyzed using Zen software (Zeiss) and ImageJ Fiji software.

### Cochlear frozen section preparation and immunofluorescence

Cochleae were sampled and fixed as previously mentioned. For immunofluorescence staining of frozen sections, the tissues were air-dried and treated with 1% Triton in PBS for 5 min, followed by incubation in blocking solution for 15 min. Then, the tissues were incubated with primary antibodies overnight at 4 °C. On the second day, the tissues were washed with PBS three times and incubated with Alexa Fluor anti-mouse/rabbit secondary antibody for 3 h. After PBS washes, the tissues were mounted with Vectorshield. Images were taken with a Zeiss 780 confocal microscope. The primary antibodies used for immunofluorescence were as follows: monoclonal anti-acetyl tubulin (1:200, Sigma, T7451, USA), rabbit anti-myosin VIIa (1:500, Proteus BioSciences, Ramona, CA, USA), rabbit anti-spectrin α (1:200, D8B7, Abcam, Cambridge, UK), anti-ATP8A2 (1:200, provided by Dr. Xianjun Zhu as shown previously [6]), TMEM30A monoclonal antibody (supernatant 1:3, a gift from Dr. Robert Molday, University of British Columbia, Canada), and mouse anti-CHOP (1:200, Cell Signaling Technology, USA). Nuclei were counterstained with either propidium iodide (PI) or DAPI (1:500).

### Scanning electron microscopy

The mice were transcardially infused with 50 ml of 0.1 M PBS followed by 50 ml of a fixation solution of 4% paraformaldehyde containing 2.5% glutaraldehyde after deep anesthesia. The cochleae were quickly removed and perfused with fixation solution through the opened oval window, round window, and drilled hole in the cochlear apex. Cochleae were immersed in fixation solution overnight at 4 °C. The next day, after three rinses with 0.1 M PBS, the cochleae were decalcified with EDTA solution until they were softened. The cochleae were postfixed in 1% osmium tetroxide solution at 4 °C for 2 h. After that, the cochleae were consecutively dehydrated in 25%, 50%, 70%, 90%, 95%, and 100% ethanol for 15 min for each time in sequence at 4 °C, followed by drying using an EM CPD 300 critical point dryer with liquid CO_2_ (Leica, Wetzlar, Germany). The dehydrated tissues were sputter-coated with platinum using an EM SCD 050 instrument (Leica) and analyzed with a Auanta 250 field-emission scanning electron microscope (FEI, Brno, Czech Republic). Three specimens were examined in both the KO and control groups.

### RNA isolation and quantitative real-time PCR analysis

Total RNA was extracted using TRIzol reagent (Invitrogen, CA, USA) according to the manufacturer’s protocol. Four to six cochleae from three mice were pooled together as one sample for the following experiments. RNA purity and quantification were evaluated using a NanoDrop 2000 spectrophotometer (Thermo Scientific, USA). RNA integrity was assessed using the Agilent 2100 Bioanalyzer (Agilent Technologies, Santa Clara, CA, USA). We used a Color Reverse Transcription kit (EZBioscience, USA) to produce cDNA following the manufacturer’s instructions. Briefly, 100 ng of RNA was mixed with gDNA remover at RT for 5 min, and then, 4× RT master mix was added. The whole mix was added up to 20 µl, and the reverse transcription reaction was performed with a PCR machine at 42 °C for 15 min and 95 °C for 30 s. The products were diluted to 100 µl with ddH_2_O for the following analysis. Quantitative real-time PCR (qRT-PCR) was performed using the White Cycler 96 system (Roche, Germany) according to the manufacturer’s instructions for the 2× Color SYBR Green qPCR Master Mix (EZBioscience, USA). The observed mRNA expression was normalized to the housekeeping gene *Gapdh*, the data were analyzed using the comparative Ct method, and the fold change was compared relative to the control group. Three replicates were performed for each assay, and each experiment was repeated at least three times. The primers are listed in Additional file 1: Table S1.

### RNA library preparation and sequencing

RNA was isolated as mentioned above. Then, the libraries were constructed using the VAHTS Universal V6 RNA-seq Library Prep Kit according to the manufacturer’s instructions. Transcriptome sequencing and analysis were conducted by OE Biotech Co., Ltd. (Shanghai, China). The libraries were sequenced on an Illumina NovaSeq 6000 platform, and 150 bp paired-end reads were generated. Approximately 49.398 M raw reads for each sample were generated. Raw reads in fastq format were first processed using fastp [18], and the low-quality reads were removed to obtain clean reads. Then, approximately 48.768 M clean reads for each sample were retained for subsequent analyses. The clean reads were mapped to the mouse genome using HISAT2 [19]. The fragments per kilobase of exon per million mapped fragments (FPKM) of each gene was calculated, and the read counts of each gene were obtained by HTSeq-count [20, 21]. Principal component analysis (PCA) was performed using R (v 3.2.0) to evaluate the biological duplication of samples.

### Differentially expressed gene analysis

Differential expression analysis was performed using DESeq2 [22]. A *Q* value < 0.05 and fold change > 2 or fold change < 0.5 were set as the thresholds for significantly differentially expressed genes (DEGs). Hierarchical cluster analysis of DEGs was performed using R (v 3.2.0) to demonstrate the expression pattern of genes in different groups and samples. The radar map of the top 30 genes was drawn to show the expression of upregulated or downregulated DEGs using the R packet ggradar. Based on the hypergeometric distribution, Gene Ontology (GO), Kyoto Encyclopedia of Genes and Genomes (KEGG) pathway, Reactome, and WikiPathways enrichment analyses of DEGs were performed using R (v 3.2.0) to screen the significantly enriched terms [23, 24]. R (v 3.2.0) was used to draw the chord diagram and bubble diagram of the significantly enriched terms.

Gene set enrichment analysis (GSEA) was performed using GSEA software [25]. The analysis used a predefined gene set, and the genes were ranked according to the degree of differential expression in the two types of samples. Then, we tested whether the predefined gene set was enriched at the top or bottom of the ranking list.

### Western blotting

Cochleae from six mice were dissected out in prechilled 0.1 M PBS and grouped into one sample. Tissues were then homogenized in RIPA lysis buffer (EpiZyme, China) with a protease and a phosphatase inhibitor (EpiZyme, China). The homogenates were centrifuged at 12,600 RCF for 20 min in a precooled 4 °C centrifuge. The supernatant was collected to measure the protein concentration using a bicinchoninic acid protein assay (BCA) kit (EpiZyme, China). Protein samples (30 µg) were separated on 4–12% SDS-polyacrylamide gels and transferred onto 0.45 μm nitrocellulose membranes. Then, the membranes were blocked with 5% nonfat milk for 1 h on a shaking table at room temperature. After this, the membranes were incubated with the primary antibodies at 4 °C overnight. After 3 washes with 1× TBST, the membranes were incubated with horseradish peroxidase (HRP)-conjugated secondary antibodies for 1 h at room temperature. Membranes were developed with enhanced electrochemiluminescence (ECL) solution (EpiZyme, China), imaged with a chemiluminescence imaging system (Bio-Rad), and analyzed using ImageJ software (National Institutes of Health, Bethesda, MD, USA).

The following antibodies were used for western blotting: TMEM30A monoclonal antibody (supernatant 1:3, a gift from Dr. Robert Molday, University of British Columbia, Canada), rabbit anti-BiP (1:1000, Cell Signaling Technology, USA), mouse anti-β-actin (1:1000, Cell Signaling Technology, USA), HRP-conjugated Affinipure Goat Anti-Mouse IgG (H+L) (1:10,000; ProteinTeck, China), and HRP-conjugated Affinipure Goat Anti-Rabbit IgG (H+L) (1:10,000; ProteinTeck, China).

### Statistical analyses

All values were expressed as the mean ± SEM. Two-way ANOVA was used for the analysis of the ABR data between groups at various evaluation timepoints. One-way ANOVA followed by post hoc test was used for the remaining experiments. Statistical analyses were performed with SPSS (Version 26.0, Chicago, IL, USA). A *p*-value < 0.05 indicated statistical significance.

## Results

### TMEM30A and P4-ATPases are expressed in a temporal- and spatial-dependent pattern in the mouse cochlea

**Fig. 1:**
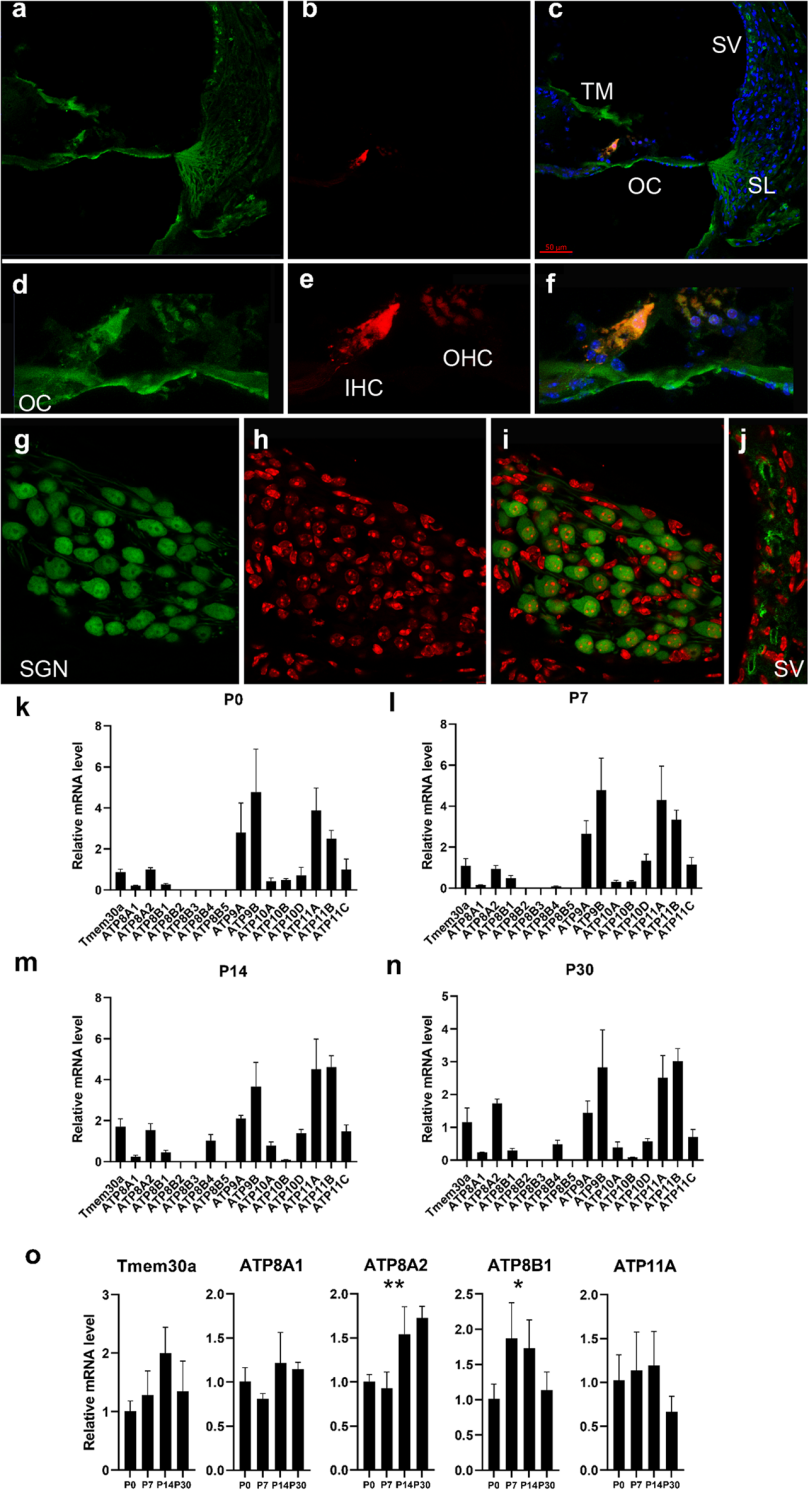
Temporal and spatial expression of P4-ATPases and TMEM30A in cochlea. Expression of TMEM30A in the inner hair cells and outer hair cells in the organ of Corti. **a**–**f** spiral ganglion neurons (**g**–**i**) in Rosenthal’s Canal and stria vascularis (**j**) in adult mouse cochlea. Enlarged organ of Corti was seen in **d**–**f**. Green TMEM30A, Red MyosinVIIa (**a**–**f**), Red propidium iodide (PI) (**g**–**j**), and blue DAPI. Relative mRNA level of P4-ATPases and *Tmem30a* at P0 (**k**), P7 (**l**), P14 (**m**), and P30 (**n**). **o** The mRNA level of *Tmem30a*, *ATP8A1*, *ATP8A2*, *ATP8B1*, and *ATP11A* changed at different postnatal development stages. Error bars indicate the standard error of the mean. *OC* Organ of Corti, *TM* tectorial membrane, *SV* stria vascularis, *SL* spiral ligament, *IHC* inner hair cells, *OHC* outer hair cells, *SGN* spiral ganglion neurons. In **k**–**o**, *N* = 3

To verify the specific expression of TMEM30A, we used immunofluorescence on cochlear cross sections. Both inner and outer hair cells showed TMEM30A expression (Fig. 1a–f). In Rosenthal’s canal, we observed TMEM30A expression in spiral ganglion neurons (Fig. 1g–i). There was also a TMEM30A fluorescence signal surrounding the capillaries of the stria vascularis (Fig. 1j). We then used qRT-PCR to clarify the expression pattern of the 15 P4-ATPase and *Tmem30a* mRNA in the cochlea during postnatal development. *Tmem30a*, *ATP11A*, *ATP11B*, *ATP9A*,* ATP9B*, *ATP10D*, and *ATP8A2* were predominantly expressed at P0, P7, P14, and P30 (Fig. 1k–n). We compared the mRNA levels at different ages and divided P4-ATPases into four groups. Genes in the first group exhibited upregulated and then downregulated patterns, including *Tmem30a*,* ATP10A*,* ATP10D*, *ATP11A*,* ATP11B*, *ATP11C*, *ATP8B1*, and *ATP8B4*. The second group showed an increasing pattern from P0 to P30, including* ATP8A2* and *ATP8B5*. The third group had a decreased expression pattern with development, including *ATP8B2*, *ATP8B3*, *ATP9A*, *ATP9B*, and *ATP10B*. However, *ATP8A1* did not show obvious changes (data not shown).

### Knockout of TMEM30A in HCs impairs hearing onset

**Fig. 2:**
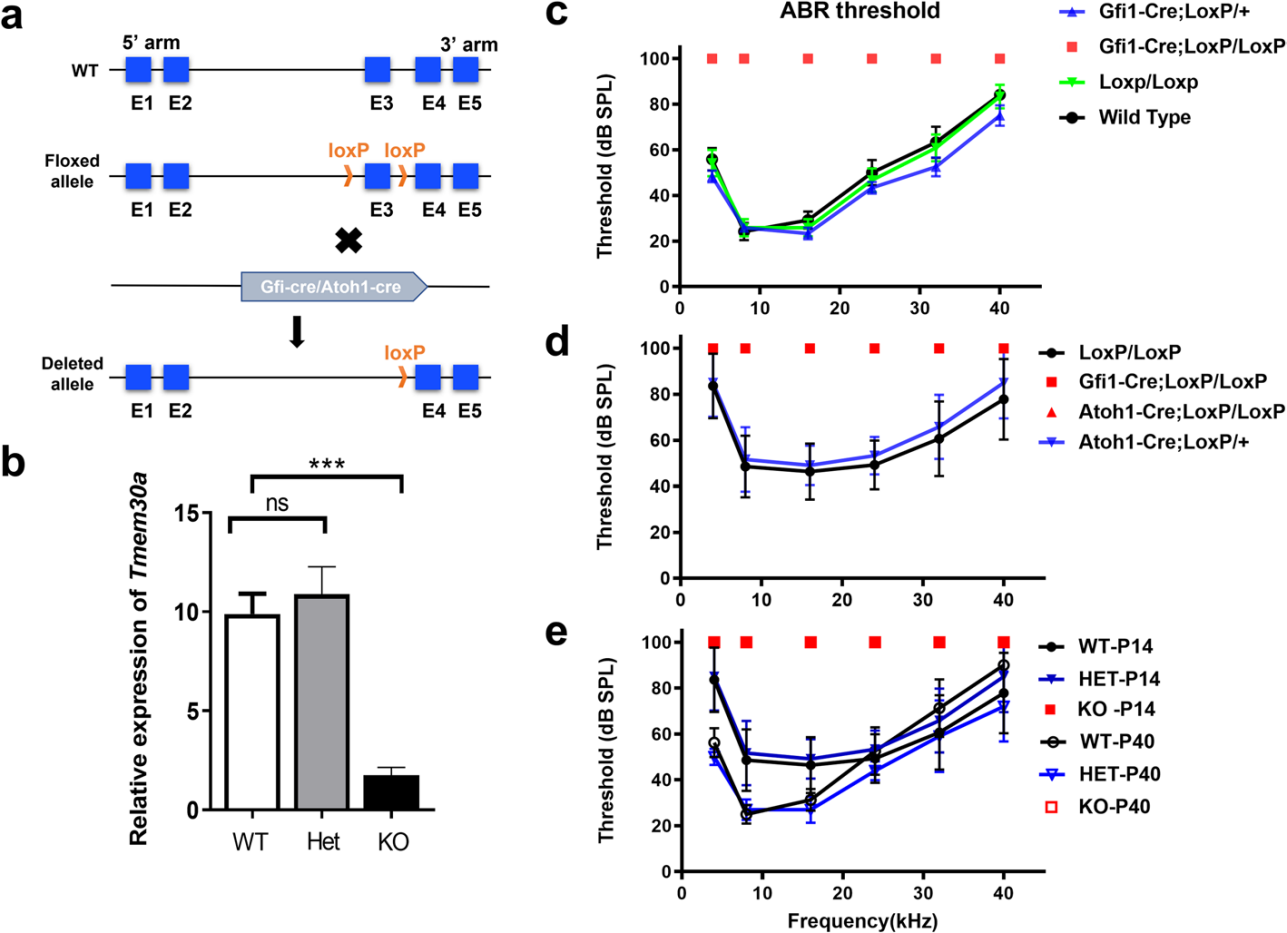
Hair cell specific deletion of TMEM30A caused hearing loss. **a** The schematic graph showed the generation of hair cell specific Tmem30a-knockout mice. Exon 3 was flanked by two loxP sites. Briefly, we generated hair cell specific deletion mice using Gfi1/Atoh1-Cre. *Tmem30a* expression was diminished to 10% in mutant cochlea, as revealed by real-time PCR (**b**). Heterozygous (Het) cochleae showed mRNA levels comparative with those in WT cochleae. **c** No detectable hearing function was shown in Gfi-Cre *Tmem30a*^Loxp/Loxp^ adult mouse, while heterozygous mice had similar ABR threshold with WT counterparts. **d** ABR results at 2 weeks old. Both Gfi1-Cre *Tmem30a*^Loxp/Loxp^, and Atoh1-Cre *Tmem30a*^Loxp/Loxp^ mice had no detectable hearing responses. **e** Auditory function improved from P14 to P40 in WT and heterozygous mice; in contrast, no detectable auditory function was shown in the KO mice. Error bars indicate the standard error of the mean. In **b** *N* = 3. In **c**–**e**, *N* = 6 or 7

To assess the effects of TMEM30A deletion on hearing function, we performed ABR analysis from the Tmem30a knockout (KO) and control mice. The schematic of TMEM30A KO mice generation was shown in Fig. 2a. *Tmem30a* expression was greatly decreased in mutant cochleae to 10% of that in wild-type (WT) cochleae, as revealed by qRT-PCR (Fig. 2b), while the expression levels were comparable in heterozygous and in WT cochleae. No detectable hearing responses were elicited in either the Gfi-Cre *Tmem30a*^loxp/loxp^ or Atoh1-Cre *Tmem30a*^loxp/loxp^ adult mice, while heterozygous mice had normal hearing function (Fig. 2c, d). We further tested auditory function at P14, which is the time of hearing onset, but no reactions were elicited (Fig. 2e). Knockout of TMEM30A in HCs affects auditory function formation, suggesting that the impairment occurred in the early postnatal stage before hearing onset.

### Progressive HC loss caused by TMEM30A deletion leads to hearing loss

**Fig. 3:**
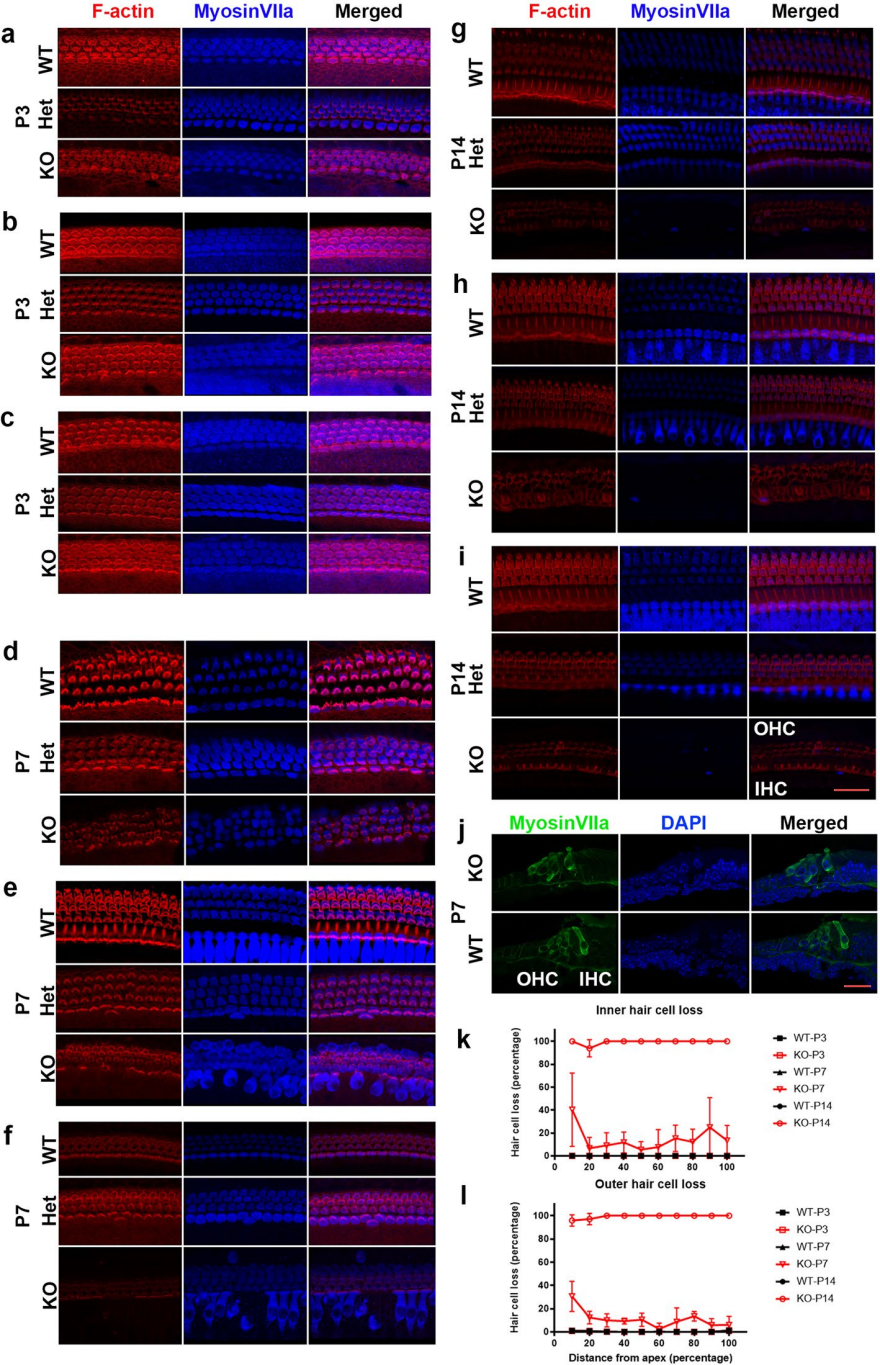
Progressive hair cell impairment during postnatal development. Hair cell staining on whole-mount preparation of basilar membrane from P3 (**a**–**c**), P7 (**d**–**f**), P14 (**g**–**i**) cochleae of WT, Het, and KO mice. Figures of each timepoint included apical, middle, and basal turn, from top to bottom. At P3, both IHCs and OHCs were intact in KO mice. Scattered HC loss was seen at P7, with more severe lost in the apical turn in KO mice. There were rarely any HCs left in KO cochleae, while the HCs were relatively normal in Het cochleae. Red shows F-actin, blue shows myosinVIIa. **j** Comparison of the hair cells in KO and WT mice on cross section. The bodies of HCs were rounded at the bottom and an obvious narrowed “neck” was seen on each HC between the cuticular plate and cell body. Green shows myosin VIIa, blue shows DAPI. **k**, **l** Quantitative analysis of IHCs (**k**) and OHCs (**l**) at different developmental stages. Scale bar in **i**, 30 μm (for **a**–**i**). Scale bar in **j**, 15 µm. Error bars indicate the standard error of the mean. In **k**, **l**, *N* = 4

We further investigated HC morphology at P3, P7, and P14 on whole mount preparations and cross sections, and the remaining HCs were quantified. We used myosin VIIa and F-actin to label hair cells and the stereocilia. At P3, both inner hair cells (IHCs) and outer hair cells (OHCs) were still intact at the apical, middle, and basal turns in the TMEM30A knockout mice (Fig. 3a–c). The morphological features of HCs were relatively comparable with those in wild-type and heterozygous cochleae. At P7, HC loss occurred at all three turns, while loss at the apical turn was most severe (Fig. 3d–f). On average, 18.69 ± 7.03, 8.32 ± 2.99, and 16.49 ± 3.91 IHCs were lost per hundred cells in the apical, middle, and basal turns. The average OHC losses were 17.61 ± 3.59, 7.45 ± 1.60, and 8.54 ± 1.93 per hundred cells in the apical, middle, and basal turns, respectively (Fig. 3k, l). At P14, there were rarely any HCs left in the knockout cochleae, while they remained intact in the heterozygous and wild-type cochleae (Fig. 3g–i). Interestingly, HCs exhibited a more rounded shape at the bottom, especially in the middle turn (Fig. 3e), and irregular alignment of HCs in the apical and middle turns was observed at P7. From the cochlear cross section, we observed that the HCs changed from a cylinder shape to a “vase-like” structure, with a round bottom and a narrow neck between the cuticular plate and cell body (Fig. 3j). The dense myosin VIIa-labeled cuticular plate blebbed out of the IHCs. These results suggest that HCs started to undergo degeneration around P7.

### Knockout of TMEM30A disturbed the alignments of stereocilia bundles

**Fig. 4:**
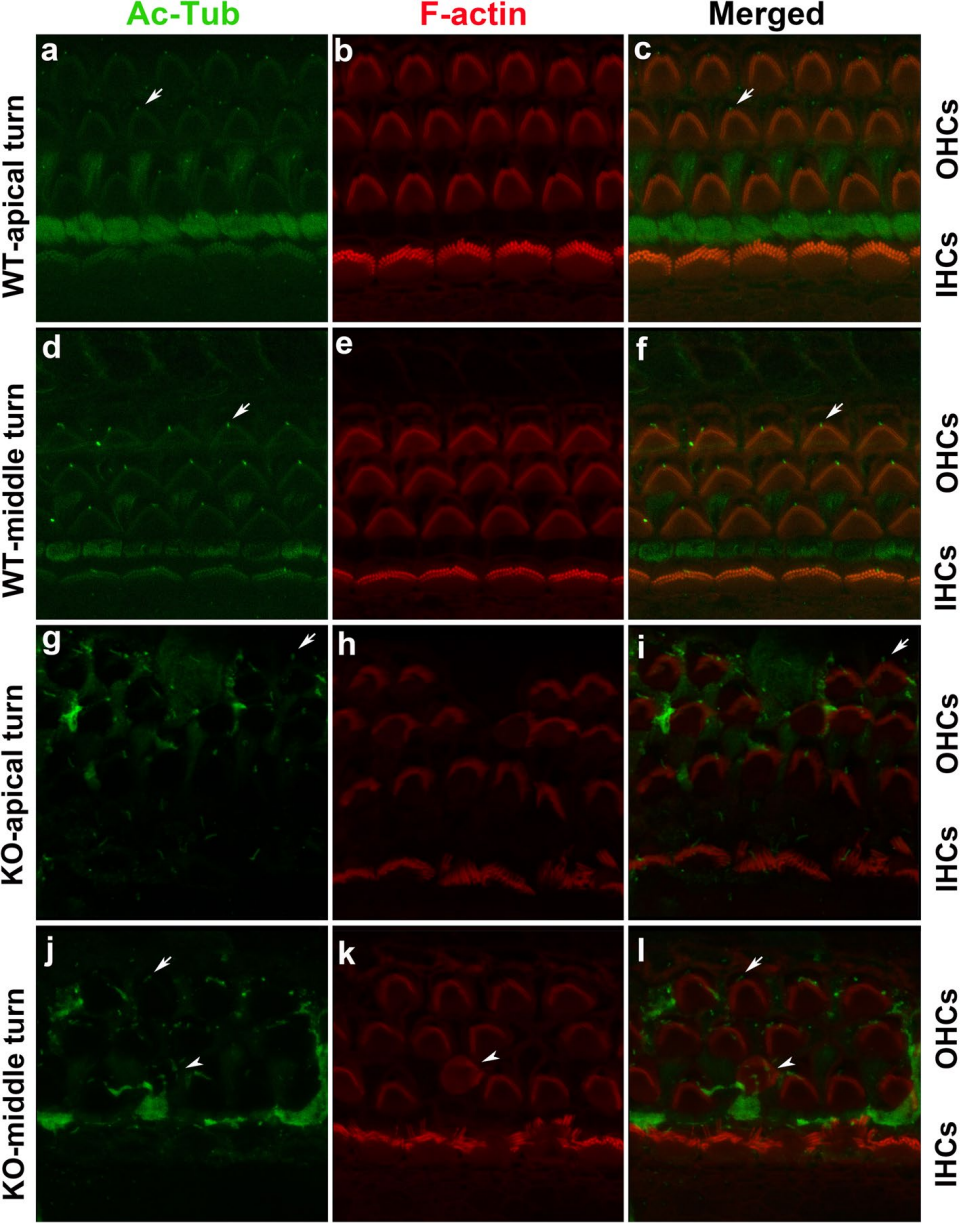
Disarrangement of kinocilia and stereocilia in TMEM30A knockout HCs. **a**–**f** Three rows OHCs and one row IHCs were distinguishable in WT cochlea. The kinocilia indicated by Ac-Tub were on top of the “V-shaped” or “W-shaped” stereocilia (arrows) in OHCs. In the middle turn, the kinocilia were seen in the center of the stereocilia where the small dents were in IHCs. **g**–**l** Scattered OHC loss was seen in the apical and middle turn of TMEM30A KO mice. The stereocilia on remaining OHCs lost the characteristic V-shape pattern. Some OHCs were disorientation (arrow head). Kinocilia were hardly recognizable, especially in IHCs. Green shows Ac-Tub, red shows F-actin

During postnatal development, kinocilia were present until P8–10. They were tidily aligned at the vertices of each stereocilia triangle, with a “V-shaped” hair bundle tip toward the lateral edge of OHCs on the cuticular plate (Fig. 4a–f) at P7. The stereocilia were stair-like and well-oriented, with the longest stereocilia toward the outer edge of HCs in WT. Notably, kinocilia of OHCs were off centered in knockout mice. The irregular and poorly defined kinocilia led to the disrupted alignment of stereocilia (Fig. 4g–l). Compared with the two- to three-row stair-like stereocilia in the WT IHCs (Fig. 4a–f), hair bundles exhibited bushy structures with no order in the KO IHCs (Fig. 4g–l). HC carries divergent/bifurcated hair bundles on the cuticular plate. Some HCs lost their original location, with disrupted polarization (arrowhead in Fig. 4j).

**Fig. 5:**
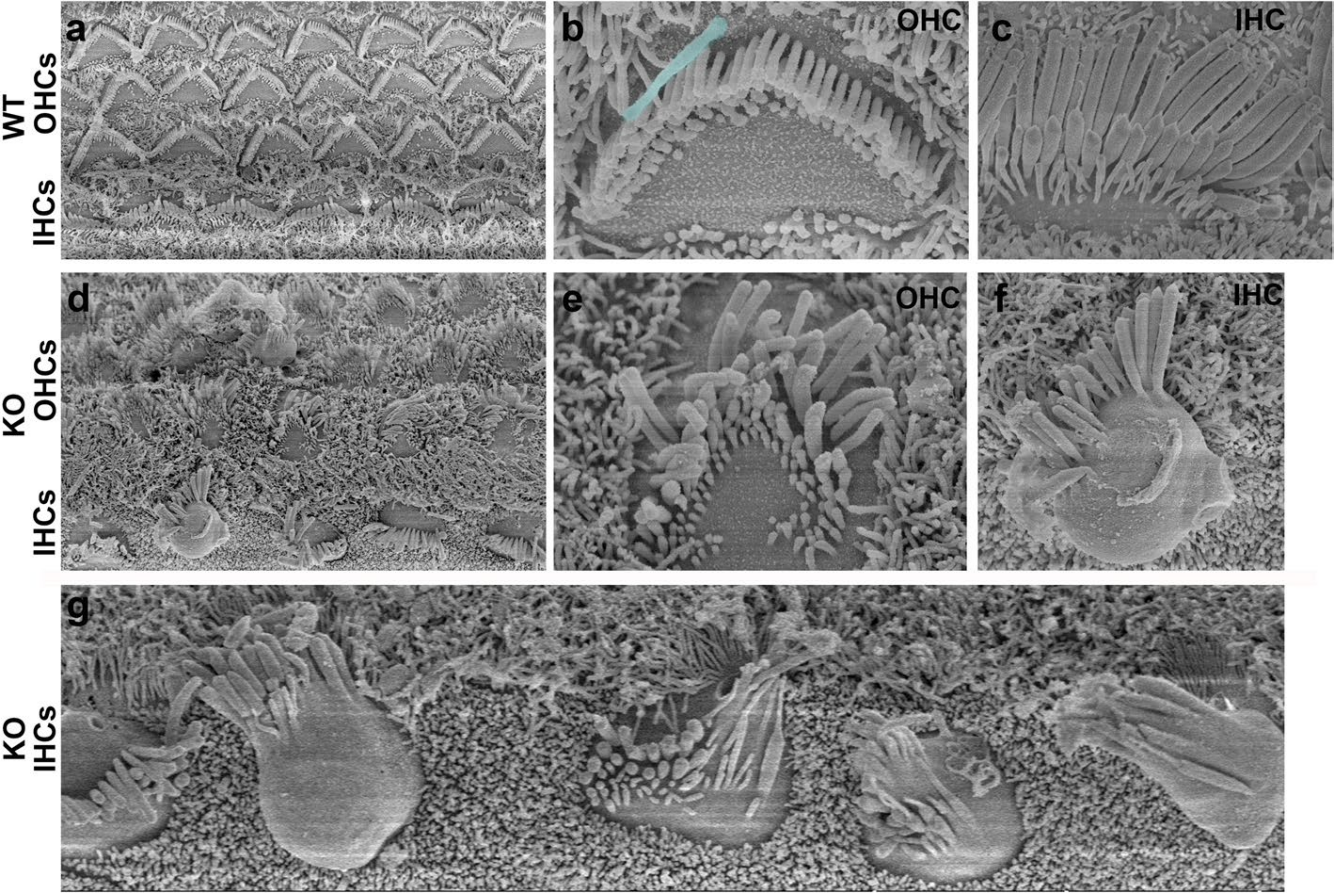
Aberrant stereocilia and undistinguishable kinocilia in IHCs and OHCs on SEM. **a**–**c** IHCs and OHCs at postnatal day 7 showing the uniform stereocilia heights and diameters within each row of the hair bundle. Kinocilium was pseudo colored in the OHC (**b**). In TMEM30A deletion HCs, the hair bundles were disorderly arranged and lodging to different directions (**d**). More than three rows of stereocilia were seen on OHCs, without recognizable kinocilium (**e**). From the top view of the IHCs, the cuticular plates were round and irregular in KO, instead of the long uniform alignment shown in WT (**a**, **c**). Stereocilia of IHCs lost their regular lodging pattern (**f**, **g**) with some located on the OHC side, some on the SGN side, and some scattered distributed without any order (**g**). Irregular arrangement of hair bundles was presented with tapered end. Samples were taken from apical turn of the cochlea

SEM images showed cell planar polarity defects in most outer hair cells (Fig. 5d). The tip of distorted “V” bundles of OHCs failed to point toward the lateral edge of the cochlea. Compared with the WT IHCs, in which kinocilia were clearly defined (Fig. 5a), stereocilia presented with no order in the KO IHCs, nor did we observe recognizable kinocilia (Fig. 5d, f, g). Even in the relatively well-organized OHCs, the stereocilia showed an immature pattern with no obvious kinocilia (Fig. 5e) compared with those in the WT (Fig. 5a, b). Extruding cuticular plate surfaces were observed in OHCs, consistent with the narrowed neck and apical blebbing of HCs, as shown on the cross section (Fig. 3j). TMEM30A KO mice exhibited disorganized stereocilia in IHCs and OHCs. Therefore, TMEM30A is proposed to be involved in the maintenance of planar cell polarity.

### Disrupted planar cell polarity caused by the deletion of TMEM30A

**Fig. 6:**
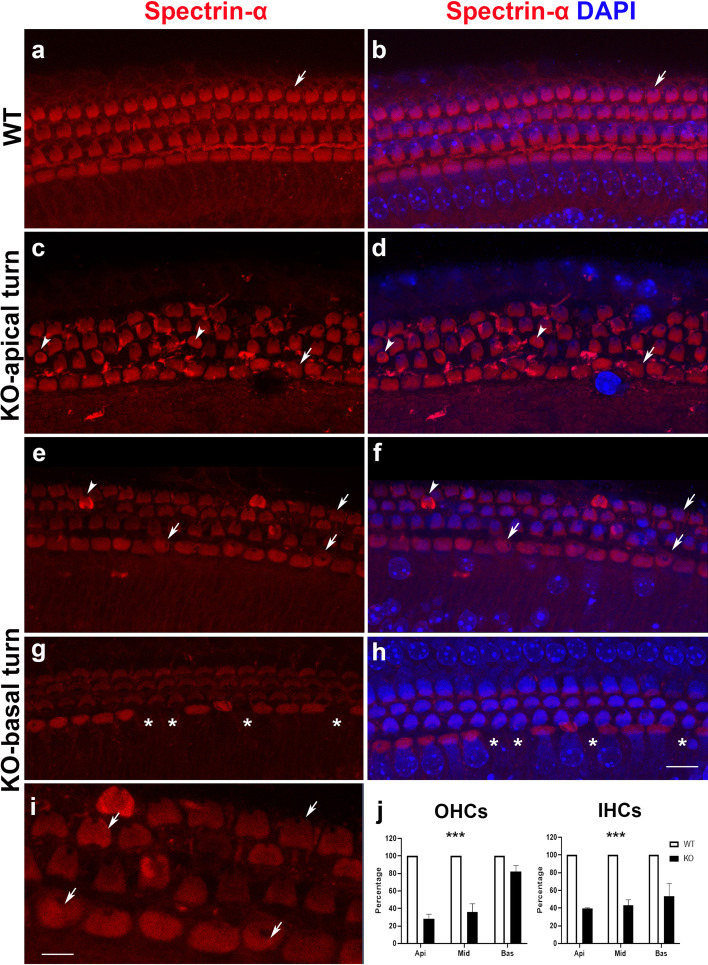
Disrupted structure of cuticular plate after TMEM30A knockout. **a**, **b** Regular alignment of cuticular plate on IHCs and OHCs in WT. A small hole (arrow) on the specific top center of the cuticular plate of each OHC indicates the location of kinocilium. **c**–**i** Disordered cuticular plates. Cuticular plate layers present with dense spectrin-α staining (arrow head) or irregular kinocilia “hole” location, which indicates disturbed cell polarity in TMEM30A KO mice. It is more severe in the apical turn than in the basal turn. From the enlarged view, we can see the randomly distributed holes on IHCs (**i**). **j** Quantitative analysis of HCs with regular polarity suggested a more severe disruption in apical turn at P7 for both IHCs and OHCs. Red spectrin-α, blue DAPI. Error bars indicate the standard error of the mean. Scale bar in **h**, 10 μm (for **a**–**h**). Scale bar in **i**, 5 μm (for **i**). We analyzed the differences of the percentage of HCs with regular polarity between WT and KO groups. ***p < 0.001. *N* = 3

Previous studies have suggested that nonerythroid α2-spectrin (spectrin-α) surrounds the rootlets of stereocilia and is abundant in the cuticular plate [26, 27]. We used immunofluorescence staining of spectrin-α on basilar membrane. In WT, the cuticular plates had a consistent arrangement, with a vacant hole at the center of the longitudinal side for the allocation of kinocilium (Fig. 6a, b). Abnormalities in the cuticular plate were observed in the KO basilar membrane, with a more rounded shape with the uneven distribution or aggregation of spectrin-α, or an enlarged root for kinocilia at atopic location (Fig. 6c–h). Consistent with the disrupted stereocilia and ectopic kinocilia (Fig. 4g–l), we observed that some HCs carried an atopic “root” hole for kinocilia and displayed chaotic polarity. We quantified HCs with normal planar polarity. The percentage of HCs with irregular polarity was much higher in the apical and middle turns than in the basal turn for OHCs. In IHCs, there were also tonotopic differences, but not significant among the three turns (Fig. 6j). In summary, deletion of TMEM30A caused irregular cuticular plate and disrupted planar cell polarity in HCs.

### Accumulation of ATP8A2 in HCs was observed after TMEM30A knockout

**Fig. 7:**
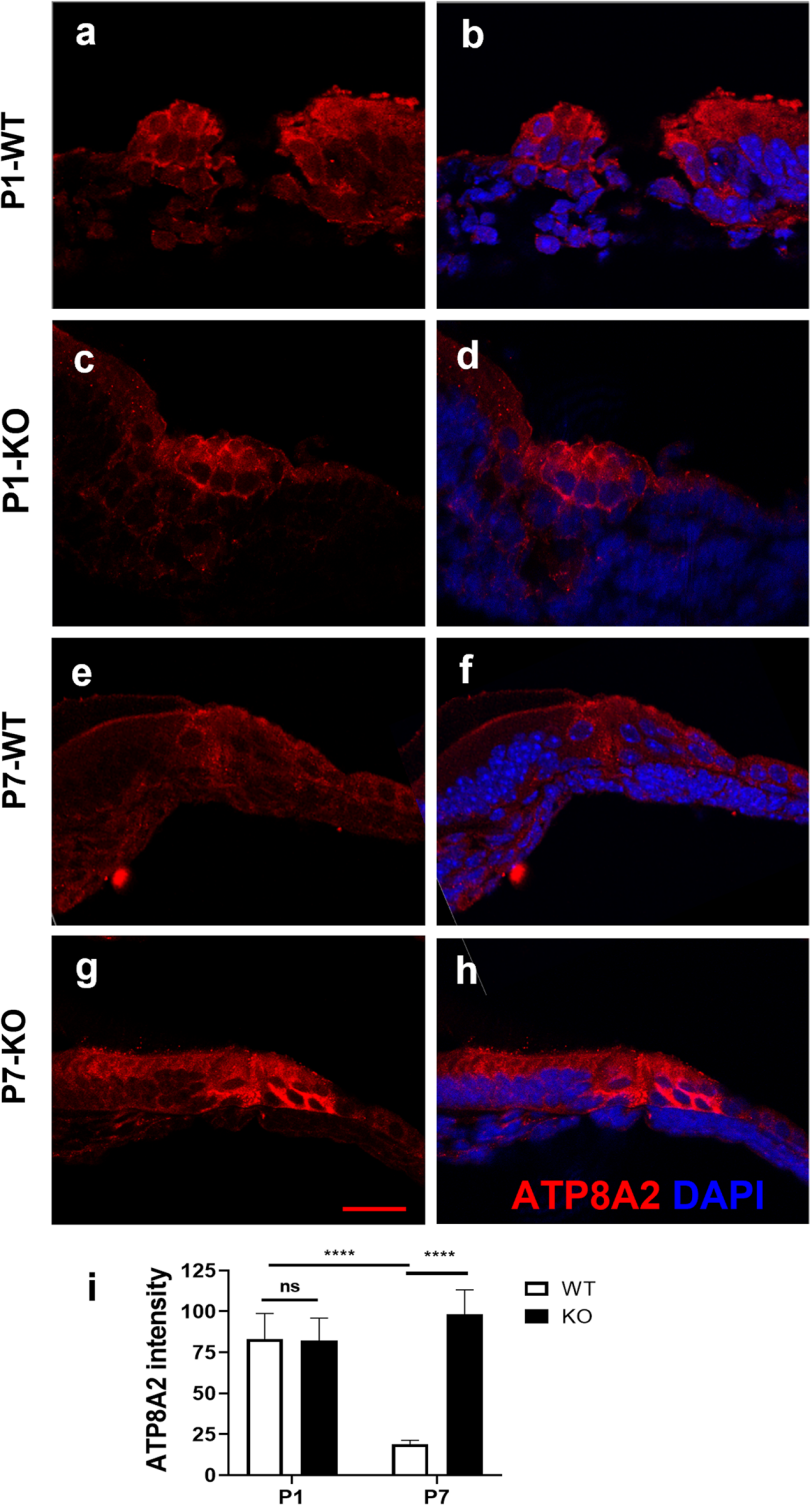
Aggregation of ATP8A2 in the HCs of P7 mouse organ of Corti. **a**–**d** At P1, ATP8A2 distributed in the cytoplasm of HCs in organ of Corti in both WT (**a**, **b**) and TMEM30A KO (**c**, **d**) mice. **e**, **f** ATP8A2 expression in cytoplasm diminished and more concentrated at the cuticular plate in WT organ of Corti at P7. **g**, **h** In TMEM30A KO cochlea, the ATP8A2 pattern was similar to that in P1 cochlea, suggesting the failed translocation to the proper subcellular structure without TMEM30A. **i** Quantitative analysis of ATP8A2 fluorescence intensity in hair cell cytoplasm. Red ATP8A2, blue DAPI. Scale bar, 20 µm. Error bars indicate the standard error of the mean. In **i**, *N* = 5 or 6

To verify how the deletion of TMEM30A affects flippases, we evaluated the expression of ATP8A2 in the organ of Corti in cross sections. ATP8A2 was highly scattered in the cytoplasm of HCs at P1. At P7, the expression of ATP8A2 in the cytoplasm decreased to approximately one-fifth of that at P1, with the TMEM30A chaperone (Fig. 7a, b, e, f). However, without the β-subunit TMEM30A, ATP8A2 was retained in the HC cytoplasm from P1 to P7, without reduction (Fig. 7c, d, g, h). Quantitative analysis indicated comparable expression of ATP8A2 in the HC cytoplasm in the KO HCs at P1, and the accumulated fluorescence of TMEM30A in the KO HCs was 5.19-fold that in the WT HCs at P7 (*p* = 6.63544E × 10^−6^) (Fig. 7i). These results indicated the accumulation of ATP8A2 with the deletion of TMEM30A.

### TMEM30A deletion and ATP8A2 accumulation led to excessive unfolded protein response (UPR) activation

**Fig. 8:**
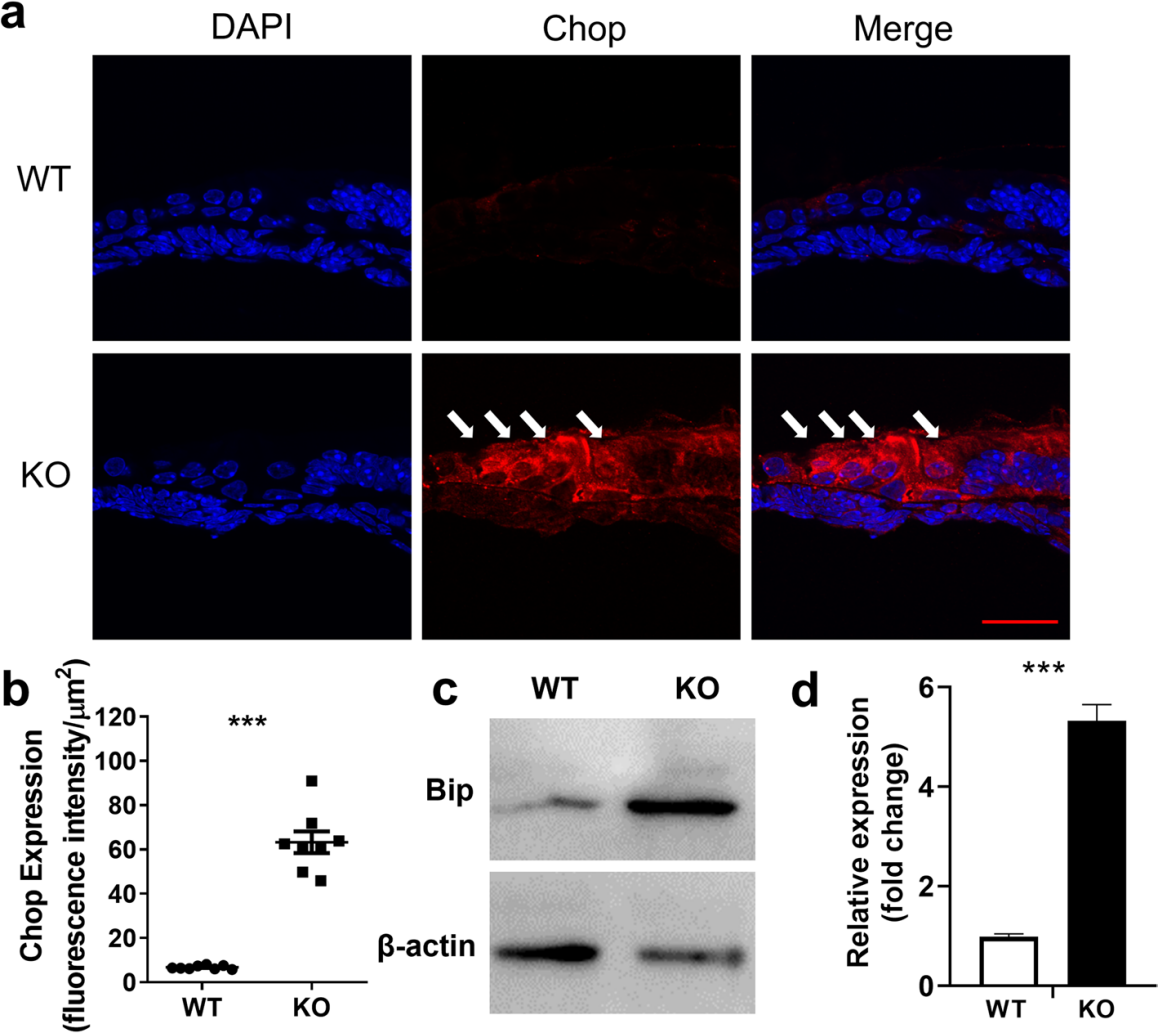
TMEM30A deletion led to increased ER stress and UPR in cochleae. **a** CHOP was absent in organ of Corti in WT mice by immunofluorescence staining on frozen section, but was accumulated in KO mice at P7. Quantitative analysis suggested about ten-fold increase of CHOP expression in KO cochleae. Red CHOP, blue, DAPI (**b**). **c**, **d** Western blotting of Bip protein indicated a five-fold increase in KO cochlea. Scale bar, 15 µm. Error bars indicate the standard error of the mean. In **b**, *N* = 8. In **d**, *N* = 3

Accumulated proteins will lead to unfolded protein responses in the ER. To examine the effects of excessive ATP8A2 on ER stress in HCs, we analyzed the critical transcription factor associated with ER stress-induced apoptosis and C/EBP homologous protein (CHOP) levels in the cochlea at P7 by immunofluorescence and immunoblotting. We observed intense fluorescence of CHOP in HCs and pillar cells in TMEM30A knockout organ of Corti, while it was almost invisible in the WT (Fig. 8a). The quantitative analysis indicated an 8.4-fold increase in CHOP expression in the knockout tissue. In addition, the expression of binding-immunoglobulin protein (BiP), also known as glucose-regulated protein 78 (Grp78), was increased 5.4-fold after deletion of TMEM30A.

### The focal adhesion gene profile changed after knockout of TMEM30A

**Fig. 9:**
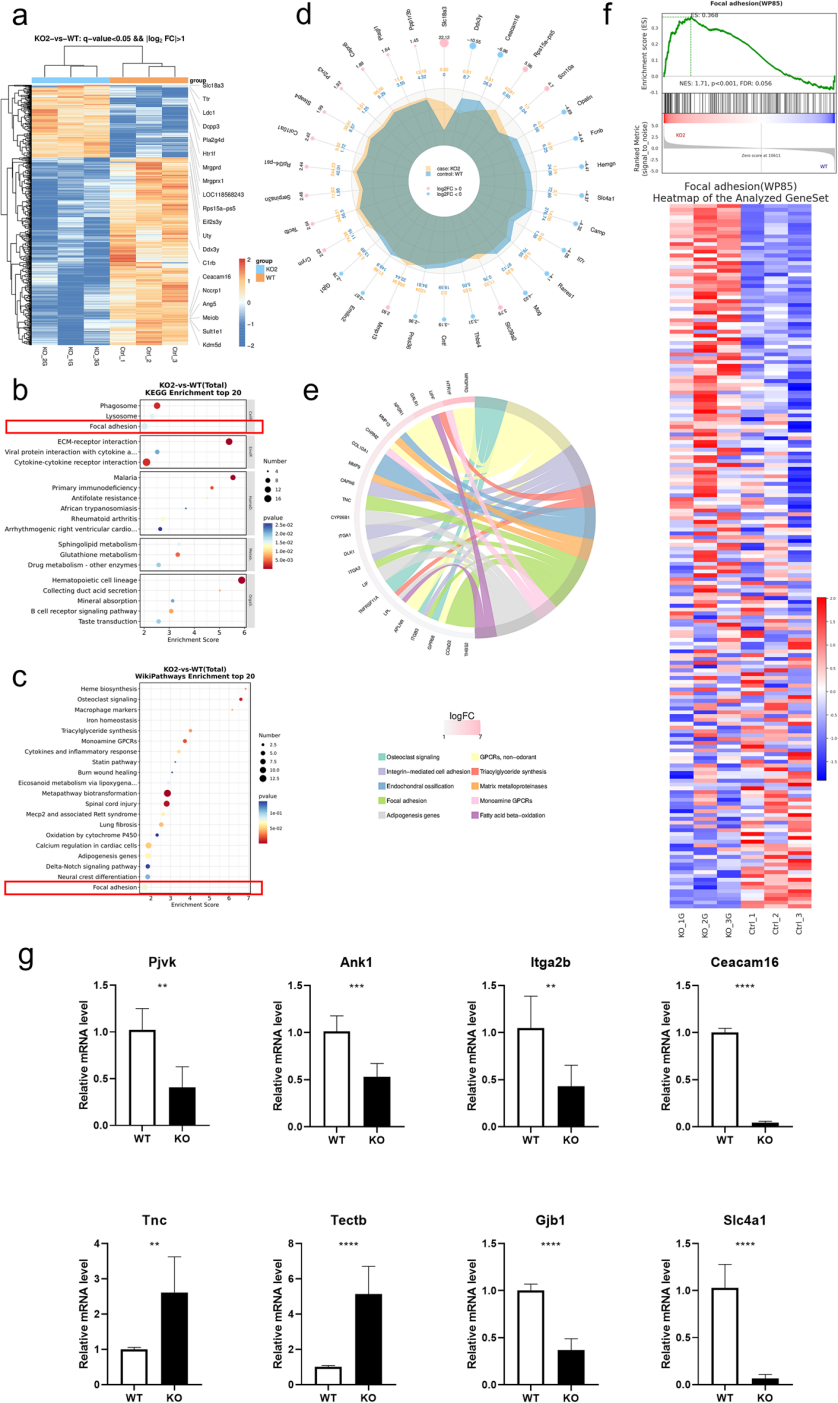
Differential gene expression profile and GO terms of up- and downregulated pathways were analyzed. **a** Heatmap of differentially expressed genes that were at least 1.5-fold up- or downregulated with the *q* value less than 0.05. **b**, **c** The top 20 most enriched KEGG and WikiPathway terms. **d** The top 30 differentially expressed genes with smallest *q* value. The genes were listed as the outer layer, with pink dots indicating upregulated genes and blue dots indicating downregulated genes. The orange color in the inner circle indicated gene profiles in KO group, the blue one indicated those in WT control group. **e** Chord chart of the most significantly different expressed genes and the top ten GO term with the smallest *p* value. **f** GSEA analysis of the focal adhesion pathway. **g** qRT-PCR analysis of the different expression genes. *GO* gene ontology, *GSEA* gene set enrichment analysis, *KEGG* Kyoto Encyclopedia of Genes and Genomes. Error bars indicate the standard error of the mean. In **g**, *N* = 3

To discover the altered regulation of gene expression after the deletion of TMEM30A, we compared the transcription profiles of the cochleae between the KO and WT mice at P7, immediately before the degeneration of the vast majority of HCs. In brief, 643 genes were differentially expressed, including 179 upregulated and 464 downregulated genes (Fig. 9a). Next, we attempted to discern the cellular component and biological process differences. We listed the top 20 enriched pathways (Fig. 9b, c). The focal adhesion pathway stood out in all three different analyses. ECM-receptor interactions, sphingolipid metabolism pathways, integrin-mediated cell adhesion, and matrix metalloproteinases were also highly enriched. We showed the 30 most significantly up/downregulated genes in the radar chart, including *Ceacam16*, *Opalin*,* Slc39a2*, *Rps15a-ps5*, *Slc4a1*, *Cntf*, *Tectb*, *Gjb1*, *Col10a1*, and *Capn6* etc. (Fig. 9d, Additional file 1: Table S2). We used the chord chart to list the top genes with the largest logFC, and the top GO terms with the smallest *p* value. Interestingly, four genes were enriched in the term “integrin-mediated cell adhesion” (*Itgb3*,* Itga2*, *Itga1*, and* Capn6*), and five genes were classified in “focal adhesion” (*Tnc*, *Itga2*, *Itgb3*, *Ccnd2*, and *Thbs2*). We further performed GSEA, and the enrichment score of focal adhesion was 0.368 (*p* < 0.01, NES 1.71) (Fig. 9f). The enrichment score for the term “stereocilium tip” was 0.664 (*p* < 0.001, NES −2.15), and *Ceacam16*, *Lhfpl5*, and* Espn* were the top downregulated genes in this group (Additional file 1: Fig. S1). We validated 15 genes by qRT‒PCR. *Pjvk*, *Ank2*, *Itga2b*, *Ceacam16*, *Gjb1*, and *Slc4a1* were downregulated, and *Tnc* and* Tectb* were upregulated, consistent with the RNA-seq results. No significant differences were observed for the genes *Tfg*, *Lhfp15*, *Mia2*, *Preb*, *Sec16b*, *Sec23*, and *Slc18a3*, but a similar trend of reduction was observed for *Lhfp15*,* Preb*, and* Mia2 *(Additional file 1: Fig. S2).

## Discussion

Our study highlighted the pivotal role of TMEM30A in maintaining the integrity and functional maturation of hair cells during postnatal development in mice and uncovered the potential mechanisms of P4-ATPase mutation-related genetic hearing loss [4, 6]. By observing the spatial and temporal expression of P4-ATPase subtypes and the β-subunit TMEM30A in the mouse cochlea, we demonstrated the expression differences of these flippases and defined the temporal pattern of TMEM30A expression in the cochlea. We also observed absent kinocilia, aberrant stereocilia arrangement, and chaotic orientation at P7, indicating that the loss of TMEM30A resulted in disrupted planar cell polarity in HCs. The accumulation of ATP8A2, which fails to translocate to the plasma membrane, leads to excessive ER stress, which subsequently causes HC death. Our results verified that the deletion of TMEM30A caused progressive HC death, with excessive ER stress, based on morphological and molecular alterations.

Asymmetric localization of phospholipids on the cellular membrane is a characteristic of eukaryotic cells. The asymmetry of the biological membrane is coordinated by P4-type phospholipid transferases. The 14 subtypes of P4-ATPases are differentially expressed on either plasma membrane or membrane bound organelles in various tissues. The function includes, but is not limited to, phospholipid asymmetry maintenance, lipid scavenging, motors in vesicle formation, and membrane scaffolding [28]. However, the detailed underlying mechanisms are not clear. TMEM30A, TMEM30B, and TMEM30C genes were mapped to human chromosomes 6q14.1, 14q23.1 and 3q12, respectively. The function of TMEM30B in the auditory system is also worth discussing in the future.

Deletion of TMEM30A, as the chaperone for P4-ATPases, has been verified to cause dysfunction in several systems [10, 29–33]. TMEM30A is highly expressed in brain tissue, followed by expression in testis, lung, vessel, kidney, and retina. The roles of TMEM30A in retinal development, bile salt transportation, and hematopoietic cell survival have been examined previously [10, 29, 30]. Conditional knockout of TMEM30A in endothelial cells impairs vessel barrier integrity by inhibiting VEGF-induced signaling to reduce proliferation. In addition, TMEM30A is essential for insulin maturation and secretion, lymphomagenesis, and skeletal muscle regeneration [31–33]. This molecule has been identified as a potential therapeutic target for endosomal anomalies in Alzheimer’s disease [34]. Hearing onset in mice occurs at postnatal days 12–14. In this study, we used both Gfi1- and Atoh1-driven Cre mice to generate a HC-specific TMEM30A knockout model [35–37]. Atoh1 is a critical transcription factor for HC differentiation and maintenance. This molecule is a master regulator of HC fate, while its overexpression generates ectopic HCs or induces supporting cell transdifferentiation into HCs [38, 39]. Not surprisingly, knockout of TMEM30A in HCs caused hearing loss. Both Gfi1- and Atoh1-Cre-driven Tmem30a knockout mice exhibited a similar phenotype. Embryonic HC development was not affected, as the KO mice carried normal HCs at P3, but HC loss was obvious starting from P7, which mimics the progressive hearing loss phenotype caused by ATP8A2 and ATP11A mutations [4, 6, 7]. Auditory dysfunction is attributed to progressive HC loss starting from P7 and peaking at P14, with only scattered HCs remaining. We found that TMEM30A is also predominantly expressed in the stria vascularis (SV). Given the previous research that deletion of TMEM30A in endothelial cells impaired vessel barrier integrity [40], exploration of its physiological function in SV would be of interest, in that the blood-labyrinth barrier in SV strictly controls precise exchange of substrate in and out of the endolymph, which maintains the stable endolymph environment for normal auditory function [41].

An interesting finding is that planar cell polarity in HCs is impaired in the KO mice. Briefly, the function of planar polarity is to maintain the accurate stereocilia arrangement on the cuticular plate, which is allocated on top of the HCs. The cuticular plate is the stiff structure in the apical region of HCs, supported by an actin mesh network. The deflection of hair bundles implements the initial step of HC polarization. HC polarity disruption deteriorates progressively in later postnatal developmental stages after TMEM30A deletion. Consistent with previous research, disrupted planar cell polarity leads to auditory dysfunction [42, 43]. Spectrin is a major component of the membrane skeleton, and its interactions with phospholipids play an essential role in the maintenance of membrane integrity and its mechanical properties [44–46]. In the HCs, spectrin-α is abundantly expressed in the cuticular plate at early postnatal stages and forms a ring-like structure around the stereocilia rootlets to maintain the regular function of stereocilia [26]. In our study, disorganized spectrin-α protein was observed and further led to the disruption of the stereocilia and cuticular plate, which is comparable to our previous research indicating the loss of stereocilia orientation in spectrin-α2 KO mice. Spectrin-α is also involved in cell adhesion and intercellular contact [47].

Another direct evidence of the disrupted planar polarity is the irregular or absent kinocilia in the KO mice. Kinocilia are the primary cilia in hair cells that are pivotal for HC morphogenesis, by dictating the proper arrangement of stereocilia. The completion of kinocilium development is before that of stereocilia, and occurs around embryonic day (E) 15. The kinocilia serve as the “guidepost” for hair bundle planar orientation, contributing to the formation of stair-like stereocilia. Under physiological conditions, kinocilia degenerate gradually from the basal to apical turn of the cochlea after P8 and completely disappear at P12 [43, 48]. The kinocilium is derived from the axoneme of the primary cilium, which projects from the center of the apical surface, and elongates and migrates eccentrically toward the abneural edge of each HC. Without TMEM30A, the kinocilia failed to find the set position and further caused the chaotic arrangement of stereocilia. In *Saccharomyces cerevisiae*, a homolog of the mammalian TMEM30 protein Cdc50p is necessary for the subcellular localization of Bni1p and asymmetrical cell division [49]. Bni1p is a homolog of human formin-homology protein and is implicated in cell polarity control and actin remodeling. Spectrin forms a functional domain to maintain HC polarity and function, likely by dictating molecular trafficking in stereocilia [27]. As the core constituents of the hair bundles and cuticular plate, TMEM30A deletion disrupts cell polarity by affecting actin dynamics, as shown in motile cells, myoblasts, and budding yeasts [49–51]. Thus, TMEM30A plays a critical role in cell polarity control. However, the detailed mechanisms are worth further exploration.

To further understand the biological and molecular processes of TMEM30A in HCs, we compared the transcriptional profile discrepancy between the KO and WT cochleae. Focal adhesion and extracellular matrix pathways were obviously altered after TMEM30A deletion. Focal adhesion is a cell–substrate junction that anchors the cell to the extracellular matrix and forms a point of termination of actin filaments. Sequestered PS localization in the cytoplasmic leaflet caused by P4-ATPase dysfunction is known to inhibit the formation of focal adhesions [52]. Integrins act as cell-surface receptors for the focal adhesion pathway in heterodimer formation and interact with a variety of ligands to promote the formation of signaling complexes regulating F-actin accumulation. Integrin α8 is expressed in HCs from E16 and persists throughout maturation, with polar localization at the apical surface where stereocilia are forming [53]. In our study, a group of integrin and integrin-based focal adhesion genes was downregulated, suggesting that the integrin-based focal adhesion pathway was impaired and responsible for the deterioration of cell polarity disruption in HCs. Several splice forms of integrin mRNAs were expressed in HCs, including those encoding integrin subunits β1, α2, α3, α6, αv, and α8. In zebrafish, integrin α8 and protocadherin-15a form a ciliary complex in neuromast HCs, and function loss of the complex leads to dysregulation of kinocilium biogenesis [54]. Reduced integrins failed to aggregate focal adhesion kinases toward the apical surface in HCs from E16 to P0, which could be the explanation for further stereocilia disruption. The evidence suggests the possible association of focal adhesion downregulation as the reason for disrupted stereocilia in TMEM30A KO mice. In addition, several genes related to the tectorial membrane were downregulated, including *Tectb*, *Tnc*, and* Ceacam16*, and these gene dysfunctions may affect the physical properties of the tectorial membrane [55]. Several downregulated genes were directly related to stereocilium tip function, such as *Pjvk*, *Lhfp15*, *Espn*, etc. Our studies provide additional insights into biological relationships between the maintenance of the phospholipid bilayer and the maturation of stereocilia, together with the formation of planar cell polarity. However, how PS localization affects stereocilia organization and planar cellular polarization by affecting focal adhesion pathways deserves further research. Machnicka et al. reviewed the potential role of spectrin in cell adhesion and cell–cell contact, and suggested that spectrin contributed to morphogenesis by its interactions with adhesion molecules, membrane proteins, and actin [56]. Together with our results, it is speculated that spectrin participated in the postnatal HC development via regulation of cell adhesion pathway with the proper function of P4-ATPases to maintain the cellular membrane homeostasis. However, further research is required.

Another prominent finding of this study is the aggregated ATP8A2 in the HCs. Consistent with previous research in retinas, deletion of TMEM30A failed to translocate ATP8A2 to the cell membrane in HCs [10], which impeded flippase activity and disturbed phospholipid asymmetry. Extra ATP8A2 accumulates in the ER, where the protein is assembled, and further provokes the unfolded protein responses (UPR). The abnormal UPR signaling induces a series of adaptive programs to maintain proteostasis. Bip is expressed in the ER lumen and assists in proteostasis, which is involved in the folding and trafficking of secretory and transmembrane proteins. It is a key regulator induced in cells upon exposure to stressors, culminating in the aggregation of misfolded proteins. Hence, hair cells lacking TMEM30A underwent UPR to reestablish proteostasis through upregulation of Bip and the downstream transcription factor CHOP. Unlike the disrupted hair cell polarity, which might be the primary effects of TMEM30A deletion, ER stress could be the direct reason leading to the HC death, as in other diseases such as endometrial carcinoma concurrent with metabolic disorders [57]. The HCs could not handle the overwhelming ectopic P4-ATPase accumulation and further led to degeneration, consistent with the results observed in rod bipolar cells.

However, the possibility of rescuing or delaying hearing loss, and at least HC degeneration, by targeting specific components of the UPR requires further discussion. In Usher syndrome, a multigenic disease characterized by sensorineural deafness and progressive retina degeneration, mutant Chd23, Harmonin, and Myo7a proteins impaired the formation and trafficking of the complex and triggered ER stress leading to cell death [58]. ER stress has also been revealed as the proximal cause of sensory cell loss in other factor-induced hearing losses, such as bilirubin or gentamicin ototoxicity [59, 60]. Intervention of ER stress to treat hearing loss has been reported [61–64]. In addition, new therapeutic regimes have been brought out for either genetic-related or acquired hearing loss, such as gene editing or hair cell regeneration [39, 65–69]. In P4-ATPase-related genetic hearing loss, targeting specific components of ER stress could be a feasible method to delay HC degeneration, which provides a treatment time niche before severe hearing loss.

## Conclusions

Taken together, this study provides an initial step in elucidating the critical role of TMEM30A in the regulation of stereocilia organization and maintenance of the planar cell polarity of HCs of the mouse cochlea for the first time. The integrin-based focal adhesion pathway might be involved in the maintenance of stereocilia polarity. In addition, the failed transportation and accumulation of P4-ATPases results in excessive ER stress, which further leads to HC death. In the future, analysis of the interaction of the focal adhesion pathway and the formation of planar cell polarity at the molecular level would be of interest.

## Supplementary Information

**Additional file 1: Figure S1.** Transcriptome analysis of differentially expressed genes in TMEM30A KO and WT cochleae at P7. **a** A total of 643 genes were differentially expressed, including 179 upregulated and 464 downregulated genes. **b** Volcano chart indicated the differentially expressed genes. **c** GSEA analysis indicated differentially expressed genes, with the GO term “Apoptosis modulation by HSP70,” were enriched in the KO cochleae. NES = 1.67, *p* = 0.016, FDR = 0.067. **d** Heatmap of “apoptosis modulation by HSP70” related genes in TMEM30A KO (KO) and WT (control) groups. **e** GSEA analysis indicated “stereocilium tip” related genes were enriched, NES = −2.15, *p* < 0.001, FDR = 0.001. **f** Heatmap of “stereocilium tip” related genes in TMEM30A KO (KO) and WT (control) groups. *GSEA* gene set enrichment analysis, *NES* normalized enrichment score, *FDR* false discovery rate. **Figure S2.** qRT-PCR validation of differentially expressed genes. A decreased trend was seen in KO group for genes *Lhfp15*, *Mia2*, and *Preb*, while the differences were not significant. No obvious differences were seen for genes *Slc18a3*, *Sec16b*, *Sec23a*, and *Tfg*. *N* = 3 for each experiment. **Figure S3.** GO term endoplasmic reticulum exit site was differentially expressed by GESA between KO and WT cochleae at P7. *N* = 3 for each experiment. ES 0.535, NES 1.83, *p *value < 0.001. *ES* enrichment score. *NES* normalized enrichment score. **Table S1.** Primers for qRT-PCR used in this study. **Table S2.** Top 30 differentially expressed genes in KO and WT cochleae.

## Data Availability

All data generated or analyzed during this study, except the RNA-sequencing datasets, are included in this article and its additional files. RNA-sequencing datasets used and analyzed during this study are available from the corresponding author upon reasonable request.

## Abbreviations

TMEM30: Transmembrane protein 30
CDC50: Cell division control protein 50
P4-ATPase: Type IV P-tape ATPase
DFNA: Autosomal dominant nonsyndromic sensorineural hearing loss
ER: Endoplasmic reticulum
PS: Phosphatidylserine
HC: Hair cell
IHC: Inner hair cell
OHC: Outer hair cell
SGN: Spiral ganglion neuron
CHOP: C/EBP homologous protein
KO: Knockout
WT: Wild-type
ABR: Auditory brainstem response
GO: Gene ontology
KEGG: Kyoto encyclopedia of genes and genomes
DEGs: Differentially expressed genes
GSEA: Gene set enrichment analysis
Bip: Binding-immunoglobulin protein
UPR: Unfolded protein responses
ECM: Extracellular matrix
NES: Normalized enrichment score
