# Supplementary material for: TMEM30A is essential for hair cell polarity maintenance in postnatal mouse cochlea
Source: Cell Mol Biol Lett. 2023 Mar 23;28:23. doi: 10.1186/s11658-023-00437-w (PMC10035192; doi:10.1186/s11658-023-00437-w)
Supplement: Supplementary file 1 — Additional file 1: Figure S1. Transcriptome analysis of differentially expressed genes in TMEM30A KO and WT cochleae at P7. a A total of 643 genes were differentially expressed, including 179 upregulated and 464 downregulated genes. b Volcano chart indicated the differentially expressed genes. c GSEA analysis indicated differentially expressed genes, with the GO term “Apoptosis modulation by HSP70,” were enriched in the KO cochleae. NES = 1.67, p = 0.016, FDR = 0.067. d Heatmap of “apoptosis modulation by HSP70” related genes in TMEM30A KO (KO) and WT (control) groups. e GSEA analysis indicated “stereocilium tip” related genes were enriched, NES = −2.15, p < 0.001, FDR = 0.001. f Heatmap of “stereocilium tip” related genes in TMEM30A KO (KO) and WT (control) groups. GSEA gene set enrichment analysis, NES normalized enrichment score, FDR false discovery rate. Figure S2. qRT-PCR validation of differentially expressed genes. A decreased trend was seen in KO group for genes Lhfp15, Mia2, and Preb, while the differences were not significant. No obvious differences were seen for genes Slc18a3, Sec16b, Sec23a, and Tfg. N = 3 for each experiment. Figure S3. GO term endoplasmic reticulum exit site was differentially expressed by GESA between KO and WT cochleae at P7. N = 3 for each experiment. ES 0.535, NES 1.83, p value < 0.001. ES enrichment score. NES normalized enrichment score. Table S1. Primers for qRT-PCR used in this study. Table S2. Top 30 differentially expressed genes in KO and WT cochleae. [file 11658_2023_437_MOESM1_ESM.pdf]

Additional file 1.

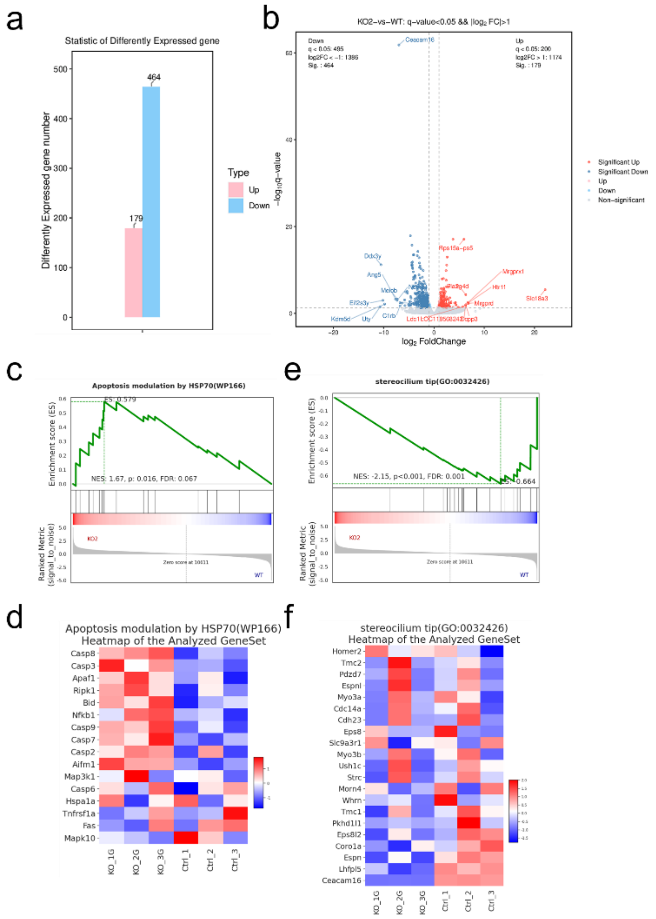

Figure S1. Transcriptome analysis of differentially expressed genes in TMEM30A KO and WT cochleae at P7. a, Totally 643 genes were differentially expressed, including 179 up-regulated and 464 down-regulated. b, Volcano chart indicated the differentially expressed genes. c, GSEA analysis indicated differentially expressed genes with the GO term “Apoptosis modulation by HSP70” were enriched in the KO cochleae. NES=1.67, *p*=0.016, FDR=0.067. d, Heatmap of “apoptosis modulation by HSP70” related genes in TMEM30A KO (KO) and WT (Ctrl) groups. e, GSEA analysis indicated “Stereocilium tip” related genes were enriched, NES=-2.15, *p*<0.001, FDR=0.001. f, Heatmap of “Stereocilium tip” related genes in TMEM30A KO (KO) and WT (Ctrl) groups. GSEA, Gene set enrichment analysis. NES, Normalized

enrichment score. FDR, False discovery rate.

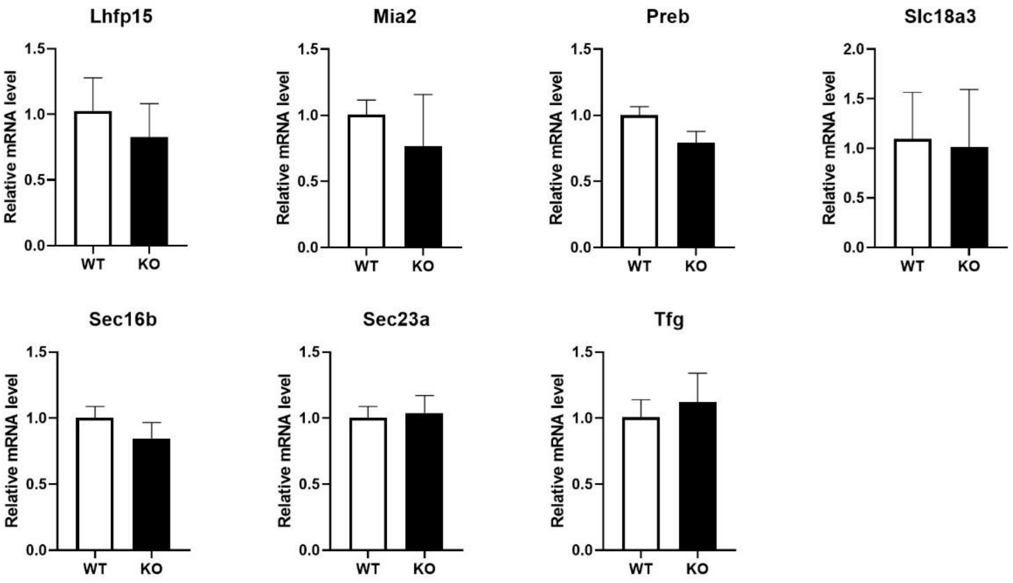

Figure S2. qRT-PCR validation of differentially expressed genes. A decreased trend was seen in KO group for genes *Lhfp15*, *Mia2*, and *Preb*, while the differences were not significant. No obvious differences were seen for genes *Slc18a3*, *Sec16b*, *Sec23a*, and *Tfg*. N=3 for each experiment.

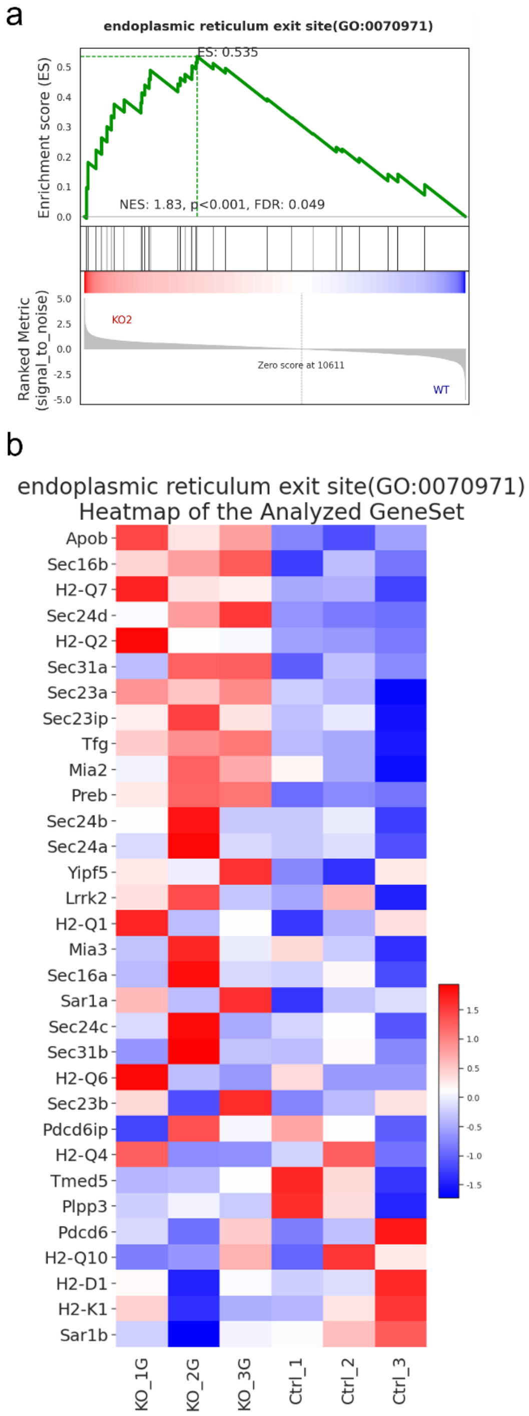

Figure S3. GO term Endoplasmic reticulum exit site was differentially expressed between KO and WT cochleae at P7 by GESA. N=3 for each experiment.

ES, 0.535, NES 1.83, with a p value<0.001. ES, Enrichment score. NES, Normalized enrichment score.

Table S1. Primers for qRT-PCR used in this study.

Gene	Forward-primer	Reverse-primer
Ceacam16	CTACGGACACATGCAGGTCTATG	CAACTGGCAGAGCGTCACCATT
Ank1	CGTGGACTCATGCTGGCATTCT	GTTCTCGGCATTCCAGGTGACT
S18a3	CGTGTACCCTTTCTAGTGCTCG	ATGAGGCGATGGATAGGTGTGC
S4a1	CTTGTGCTAGGCTTCTCAGGAC	CCACCAACATCACGAGCAGGAT
Gjb1	GTGGACCTATGTCATCAGTGTGG	GGAAGGCTTCACACTTGACCAG
Tectb	GGTCCATGAAGGTGGCTATTACC	TGTCGTTGGACACGATGTGGCT
Itga2b	GGTGCTGACAATGTGTTGGAGC	CTTCTGAGTGCAGACAAGCCTC
Pjvk	TCTGATTCCATCGCAGTCAAAGC	TGCTGCTCCTTGATTGGCGGAT
Tnc	GAGACCTGACACGGAGTATGAG	CTCCAAGGTGATGCTGTTGTCTG
Sec16b	GGACATGCCTTGTTCCTAGCCA	TGGCTGCTTGTGGAATCCTTCC
Tfg	AGAACCAGGACCTTCCACCAGT	GCTGCCATAACCTGAGTTGACTG
Preb	CAGTGGCAAGAGAATGGACCAG	GGTATCTGCACTGTGAAGAGTCG
Mia2	GAAGTGTGGTGACTTGGAATGTG	CTCCCGTAGTGAAGTTCAGGTAC
Sec23a	TGGAACAGGAGGCACTTGTCAG	GCCACTTGAGTGCTGATACTGG
Lhfpl5	GCTCCATCATCTGCTTCAGCCT	CCCAACCATCTGGGTAGACTAG

Table S2. Top 30 differentially expressed genes in KO and WT cochleae.

gene_ID	Mean expression in WT	Mean expression in KO	log2 Fold Change	q-value	Description
Ceacam16	5587.769462	44.84136957	-6.964260407	1.50E-62	carcinoembryonic antigen-related cell adhesion molecule 16
Opalin	529.1698077	20.55550229	-4.686627679	1.31E-18	oligodendrocytic myelin paranodal and inner loop protein
Rps15a-ps5	10.25475543	634.7806776	5.963318189	8.11E-18	N/A
Slc39a2	74.84184094	1031.441711	3.785693093	8.11E-18	solute carrier family 39 (zinc transporter), member 2
Slc4a1	11247.12964	542.890431	-4.372799785	1.85E-16	solute carrier family 4 (anion exchanger), member 1
Cntf	808.4329237	88.43922859	-3.193101288	5.42E-15	ciliary neurotrophic factor
Mog	5659.029798	346.9276574	-4.027459647	3.04E-14	myelin oligodendrocyte glycoprotein
Rarres1	3921.930424	228.6855597	-4.10031152	3.37E-14	retinoic acid receptor responder (tazarotene induced) 1
Thbs4	555.4440117	56.02492203	-3.307807313	4.77E-14	thrombospondin 4
Crym	482.0668707	2992.123222	2.634255684	1.08E-13	crystallin, mu
Prss36	11647.57592	1496.883963	-2.960066235	1.47E-13	protease, serine 36
Il7r	195.6567367	10.34420548	-4.245091358	1.45E-12	interleukin 7 receptor
Hemgn	1898.582708	89.12546161	-4.413538312	2.08E-12	hemogen
Emilin2	21500.78722	2937.774858	-2.871610516	2.42E-12	elastin microfibril interfacer 2
Camp	5662.919807	283.8130677	-4.318768809	6.01E-12	cathelicidin antimicrobial peptide
Ddx3y	1443.216563	0.963558563	-10.54926168	6.19E-12	DEAD box helicase 3, Y-linked
Tectb	4524.40761	26333.67964	2.541116907	7.00E-12	tectorin beta
Fcnb	425.2322311	19.53952495	-4.442919568	7.54E-12	ficolin B
Gjb1	968.3629718	141.1568367	-2.778071156	1.22E-11	gap junction protein, beta 1
Gypa	3009.964854	149.996171	-4.326831428	1.22E-11	glycophorin A
Hsd11b1	1102.731557	151.6140464	-2.862539161	2.72E-11	hydroxysteroid 11-beta dehydrogenase 1
Rhd	2360.30221	121.9514903	-4.274779838	3.34E-11	Rh blood group, D antigen
Ahsp	7846.841153	421.6703833	-4.217969978	4.71E-11	alpha hemoglobin stabilizing protein
Fam178b	2015.753035	337.8351411	-2.577163776	5.17E-11	family with sequence similarity 178, member B
Pmp2	3965.329319	903.7296817	-2.133602804	1.35E-10	peripheral myelin protein 2
Col10a1	184.35133	986.5487994	2.420462351	1.39E-10	collagen, type X, alpha 1
Ermn	1597.190968	91.6546334	-4.122087853	2.58E-10	ermin, ERM-like protein
Otop1	502.200238	64.37073557	-2.965176091	1.41E-09	otopetrin 1
Btnl10	528.8991423	38.15265592	-3.794155419	2.11E-09	butyrophilin-like 10
